# Baculovirus Surface Display of Immunogenic Proteins for Vaccine Development

**DOI:** 10.3390/v10060298

**Published:** 2018-05-31

**Authors:** Balraj Premanand, Poh Zhong Wee, Mookkan Prabakaran

**Affiliations:** Temasek Life Sciences Laboratory, 1 Research Link, National University of Singapore, Singapore 117604, Singapore; prem@tll.org.sg (B.P.); pohzhongwee@gmail.com (P.Z.W.)

**Keywords:** recombinant baculovirus, surface display, vaccine, infectious diseases

## Abstract

Vaccination is an efficient way to prevent the occurrence of many infectious diseases in humans. To date, several viral vectors have been utilized for the generation of vaccines. Among them, baculovirus—categorized as a nonhuman viral vector—has been used in wider applications. Its versatile features, like large cloning capacity, nonreplicative nature in mammalian cells, and broad tissue tropism, hold it at an excellent position among vaccine vectors. In addition to ease and safety during swift production, recent key improvements to existing baculovirus vectors (such as inclusion of hybrid promoters, immunostimulatory elements, etc.) have led to significant improvements in immunogenicity and efficacy of surface-displayed antigens. Furthermore, some promising preclinical results have been reported that mirror the scope and practicality of baculovirus as a vaccine vector for human applications in the near future. Herein, this review provides an overview of the induced immune responses by baculovirus surface-displayed vaccines against influenza and other infectious diseases in animal models, and highlights the strategies applied to enhance the protective immune responses against the displayed antigens.

## 1. Introduction

The existing battle between the human immune system and pathogens has a long-lasting history and is destined to continue. Due to the advancement in the field of infectious diseases and the discovery of effective vaccines, therapies in recent history have aided in controlling the spread of diseases. However, the looming danger of re-emerging pathogens poses a serious issue and threatens to tilt the newfound equilibrium. Among the types of vaccines available, inactivated vaccines are generally considered safe and stable. Given their inactive nature, these vaccines can be given to people with weakened immune systems without the serious complication of opportunistic infection. However, the immune responses induced by inactivated vaccines are mainly humoral, and repeated immunization is required to improve immunity to the targeted disease. Moreover, an inactivated vaccine may not retain the correct antigenic conformation and thus induces poor immune response against the targeted antigen [1]. In contrast, live attenuated vaccines are more effective in inducing protective immune responses in vaccinated individuals. The major risks of live attenuated vaccines are the possibility of reversion and recombination with circulating strains, as well as the incompatibility of the vaccine with the immunocompromised, the elderly, the chronically ill, and the pregnant [2].

Recombinant subunit vaccines, on the other hand, can generally be used regardless of health status but possess other disadvantages. Some of their disadvantages include the need for supporting adjuvants to improve immunogenicity, and purification issues due to the hydrophobic nature of antigens, which complicates the purification process, reducing the cost-effectiveness of vaccine production [3]. DNA vaccine utilizes genetically engineered DNA capable of inducing humoral and cell-mediated immune responses against parasites, bacteria, and viruses [4,5]. Modification of elements in the DNA, such as promoters or enhancers, can enhance the expression of the encoded protein in vaccine recipients. Moreover, a mixture of plasmids encoding a plethora of immunogenic genes can potentially be used to generate broad-spectrum vaccines. However, DNA vaccine may induce antibodies against DNA, resulting in autoimmune responses and the development of immunologic tolerance in recipient host [6].

Since 1985, viral vaccine vectors have emerged, and such vectors have a more favorable safety profile than live attenuated virus vaccines, while having better immunogenicity than inactivated vaccines. Viral vaccine vectors present the desired antigens in their native conformation, resulting in a stronger immunogenic response, while maintaining a higher level of foreign gene expression in vivo compared to DNA vaccines [7,8]. However, some considerations exist for the use of viral vectored vaccines in humans: (1) pre-existing vector immunity may have a serious impact on vaccine vector efficacy and (2) increased transgene size could cause genetic instability and decrease viral yield. To overcome the issues of pre-existing vector immunity, vaccine vectors utilizing recombinant viruses of nonhuman origin have been developed to avoid neutralization of viral vector by pre-existing antibodies. A class of virus infecting insect cells, known as baculoviruses, has been developed for use as nonhuman viral vectors. Among the various baculoviruses, *Autographa californica* multicapsid nucleopolyhedrovirus (AcMNPV) is the most widely studied. AcMNPV is a large, double-stranded DNA virus with a genome size of 133.9 kbp and contains 156 open reading frames (ORFs) [9]. AcMNPV has a biphasic life cycle resulting in the production of two forms of virus, budded virus (BV) and occlusion-derived virus (ODV). The ODV is responsible for the primary infection of the host insect, while BV is released from the host cells for cell-to-cell infection. Moreover, the BV is produced during early stages of infection, whereas the ODV is produced during late stages of viral infection, becoming concentrated in the nucleus and occluded within polyhedra [10]. Generally, AcMNPV expresses each gene at specific time phases during its replication cycle. The genes encoding proteins that are responsible for viral replication and/or late gene expression (e.g., *dnapol*, *lef1-12*) are expressed at an early time; structural protein genes (e.g., *vp39*, *p6.9*, and *e25*) are expressed at late times; some genes are expressed at both early and late phases (e.g., *ie1*, *pp31*, and *gp64*); and *p10* and *polyhedrin* (*polh*) are highly expressed at late and very late times of infection [11]. Based on published reports, the AcMNPV promoters *polyhedrin* (*polh*) or *p10* have been extensively used for expression of foreign proteins. In addition to the conventional AcMNPV and other viral promoters, *polh* and *p10* showed high levels of recombinant protein expression [12]. Interestingly, the combination of some of these promoters exhibited higher levels of protein expression compared to standard late promoters alone [13,14,15,16]. Moreover, it is well known that the *polyhedrin* promoter is active in the late stage of infection, and budding baculoviruses in the early stage of infection are unable to efficiently incorporate the target protein on the baculovirus envelope. Hence, the use of immediate-early promoter may improve incorporation of target protein into the viral envelope. Its salient features, including the ease of high-titer virus production, capability for simultaneous delivery of multiple genes, potential transduction ability, as well as its nonpathogenic and nonreplicative nature in humans, have led to the use of AcMNPV in the production of complex eukaryotic protein in vitro, ex vivo, or in vivo [17,18,19] and also for the vaccine development using surface display technology [19]. In this review, we describe the immune responses elicited by recombinant baculovirus displayed vaccines against various infectious diseases and explore the unique strategies used to enhance the protective immunity against baculovirus displayed antigens (Table 1).

## 2. Surface Display of Influenza Proteins

Since 1918, influenza virus has been considered as an ever-present threat to humans, causing annual epidemics and infrequent pandemics, resulting in emergence of new virulent strains that pose a sustained alarm as a public health emergency [62]. Currently, the best method for the prevention and control of influenza virus infections is via vaccinations. However, a traditional influenza virus vaccine, while effective at managing infections, faces severe challenges. Limited vaccine production capacity, long production time, poor growth properties of selected vaccine strains, and necessity of high-level biocontainment facilities are some major issues limiting vaccination production. Particularly, uncertainty in prediction of emerging pandemic viral subtypes poses an obstacle in the prompt production of vaccines during an influenza pandemic. Thus, a novel platform for the rapid development of vaccines, which overcomes limitations of currently available traditional vaccines, is required for swift response to influenza outbreak. Hemagglutinin (HA) is a major surface glycoprotein of the influenza virus and is the main target for generating protective immunity against influenza virus [63]. Known to be a key determinant of host specificity, introduction of new HA subtypes was found to be associated with influenza strains responsible for pandemic outbreaks [64,65,66,67]. Hence, HA could serve as an important ideal target for development of influenza vaccines. Recombinant subunit vaccines present one such method for targeting HA. Recombinant subunit vaccines targeting HA were developed using an insect cell expression system and have been extensively evaluated in humans and animals as influenza vaccines. Previously, a trivalent recombinant HA influenza vaccine—Flublok^®^, produced in insect cells using a baculovirus expression vector—was approved by the US FDA. However, the need for a high dose with adjuvants and vaccine purification issues due to the hydrophobic nature of HA pose major setbacks in the development of recombinant subunit vaccines. An alternative strategy in vaccine development is the use of the baculovirus surface display technique. With baculoviral vaccines, targeted protein can be expressed on the surface of the virus without affecting the replication of the virus. Moreover, insect cells possess the necessary cellular machinery to perform extensive post-translational modifications, such as glycosylation [68], phosphorylation [69], and disulfide bond formation [70], essential for the expression of many complex eukaryotic proteins, which require such modifications to maintain structural integrity and biological activities. Furthermore, it has been reported that baculoviruses have strong adjuvant properties and are capable of inducing humoral and cellular immune responses against vaccine antigens [71].

Glycoprotein 64 (GP64) is a major envelope protein of AcMNPV which is essential for viral infection in insect cells. Through the addition of GP64 domains (signal sequences, transmembrane and cytoplasmic regions), foreign proteins such as glutathione-*S*-transferase, human immunodeficiency virus (HIV) GP120 protein, rubella virus envelope protein, and synthetic IgG binding domains can be displayed on the baculoviral envelope [72,73,74]. Alternatively, certain membrane proteins can be displayed on the budded virus itself without the need for GP64 domains. For example, vesicular stomatitis virus (VSVG) protein and measles virus receptor can be independently displayed on the viral envelope [74,75]. Similarly, HA protein can be incorporated into the viral envelope without GP64 domains.

### 2.1. Recombinant Baculovirus Displayed H5 Subtype HA Protein and Its Immunogenicity

The different regions of GP64, such as the signal sequences, transmembrane domain, and cytoplasmic domain, have been widely used for the expression of secretory proteins in baculovirus/insect cell expression systems, and have been exploited to display foreign proteins or peptides on viral envelopes [76,77]. In a case study, the efficiency of H5HA incorporation into the baculoviral envelope was shown to differ between recombinant baculoviruses carrying HA with different cytoplasmic domains (CTDs), despite equal expression of recombinant HA. Bac-HA64, which expresses histidine-tagged HA with GP64-CTD (cytoplasmic domain of GP64), incorporates HA to the viral envelope more efficiently than Bac-HA, which expresses HA with HA-CTD (cytoplasmic domain of HA). Moreover, Bac-HA64 elicited higher hemagglutination inhibition titer (HI) compared to Bac-HA. The result indicates the importance of the cytoplasmic domain and the degree of palmitoylation on the domain in the incorporation of HA on the viral envelope and on vaccine potential [76]. In a second study, a modified Bac-HA64 vaccine was developed by employing baculovirus *p10* and cytomegalovirus (CMV) promoters with HA, which demonstrated surface display and endogenous expression of HA after transduction. An immunization study on the vaccine vector system revealed superior or equivalent immune responses against HA compared to other vaccine forms. The immune response is mediated by interaction with antigen-presenting cells (APCs) via the major histocompatibility II (MHC-II)-mediated antigen presentation pathway [20].

In general, vaccines targeting respiratory viral infections, like influenza, act by inducing neutralizing antibodies that neutralize virions or block viral attachment and entry into host cells. However, currently available conventional vaccines induced humoral responses against only homologous strains and provided inadequate protection against heterologous strains. In contrast, T-cell-mediated cellular immune responses against conserved internal regions of antigens mediate protective immunity against heterologous strains [21,78,79]. In previous studies, stimulation of helper T-lymphocytes (Th cells) and cytotoxic T-lymphocytes (CTLs) by nucleoprotein amino acids NP55-69 and NP380-393 provided complete cross-protection in challenge studies [22,23]. Sridhar et al. [80] observed that individuals having a higher number of pre-existing CD8^+^ T-cells against conserved CD8 epitopes showed milder illness after infection with pandemic H1N1 influenza virus [80]. Furthermore, Wilkinson et al. [81] showed that, in the absence of antibody responses, pre-existing CD4^+^ T-cells respond to influenza internal proteins, reduce the severity of illness, and demonstrate lower shedding of virus [81]. Recently, Zhang et al. [82] developed a recombinant baculovirus-based vaccine containing three tandem copies of the highly conserved extracellular domain of influenza M2 protein (M2e) (BV-Dual-3M2e); a second vaccine had an additional mucosal adjuvant heat-liable enterotoxin B (BV-Dual-3M2e-LTB). Mice inoculated with BV-Dual-3M2e-LTB vaccine induced higher levels of mucosal antibodies and more efficient cellular immunity against different H5N1 clades (clade 0, 2.3.2.1, 2.3.4, and 4) compared to non-adjuvanted BV-Dual-3M2e vaccine. Interestingly, it was discovered that mucosal immunity alone was insufficient for protection from lethal H5N1 challenge, whereas adjuvanted vaccine provided enhanced protection against similar challenge through CD8^+^ T-cell response. To validate the role of CD8^+^ T-cells in enhancing protective immunity, mice with depleted CD4^+^ or CD8^+^ T-cells were inoculated with BV-Dual-3M2e-LTB. Mice with depleted CD4^+^ T-cells displayed reduced lung viral titers, but no significant effect on survival rate was observed. Comparatively, depletion of CD8^+^ T-cells or both CD8^+^ T-cells and CD4^+^ T-cells markedly increased lung viral titers and decreased survival rate. Further research on enhancing HA-specific immune responses utilized a baculovirus vaccine vector with additional elements to improve immunogenicity. These elements included *CAG* promoter, MHC Class I signal sequence (MHCIss), MHC Class I trafficking domain (MITD), and Woodchuck hepatitis virus posttranscriptional regulatory element (WPRE). *CAG* promoter enables robust expression of HA, while MHCIss and MITD enhance MHC Class I and II antigen presentation, which stimulates the epitope-specific CD4^+^ and CD8^+^ T-cells. WPRE, on the other hand, elevates the HA gene expression and increases immunogenicity. As shown in the study, mice immunized with baculovirus possessing the additional elements induced higher levels of HA-specific IgG and IgG2a, higher hemagglutination inhibition titers, and better Th1 and IFN-γ^+^/CD8^+^ T-cell responses compared to baculoviruses without the additional elements [83]. Furthermore, the role of WPRE and ITRs (inverted terminal repeats) in enhancement of HA expression and their effect on induction of humoral and cellular immune responses was studied. Two series of baculovirus that use different promoters to drive the expression of HA were generated: BV-S series (BV-S-HA, BV-S-ITRs-HA, BV-S-con-HA), which utilizes the *CMV* promoter for the expression of HA, and BV-A series (BV-A-HA, BV-A-ITRs-HA, BV-A-con-HA), which utilizes white spot syndrome virus (WSSV) immediate-early promoter one (*ie1*) for the expression of HA. Results from immune studies indicate that baculoviral vaccine with WSSV *ie1* promoter induces a higher level of humoral and cellular immunity compared to similar baculoviral vaccine utilizing the *CMV* promoter [84]. In another study, recombinant baculovirus vectors with an *EGFP* expression cassette under *p10* and *CMV* promoters were examined. The baculoviral vectors were modified to express HA using baculovirus polyhedrin (*polh*) promoter (vAc-HA) or using dual-promoter with *polh* and *CMV* promoter (vAc-HA-DUAL). While HA was expressed efficiently in SF9 insect cells for both constructs, mice vaccinated with vAc-HA induced higher levels of IgG and IgA compared to mice vaccinated with vAc-HA-DUAL. In addition, stimulation of splenocytes in immunized mice with HA-specific peptide triggered Th2 immune response for vAc-HA-immunized mice, whereas vAc-HA-DUAL-immunized mice showed Th1-biased immune response. Moreover, vAc-HA-Dual vaccine was more effective in inducing HA-specific CD4^+^ and CD8^+^ T-cell response in vitro [24].

It has been reported that the signal peptide of membrane proteins is important in directing the protein to the endoplasmic reticulum membrane and in protein trafficking [85]. The transmembrane (TM) domain plays a role in protein trafficking, membrane anchoring, membrane fusion, and viral budding [86,87], whereas the CT domain is crucial for envelope incorporation, virus budding, interaction with the components of viral core [88,89], and incorporation of influenza HA into the baculovirus envelope [76]. Supporting these observations was the research by Tang et al. [25] on the functional role of signal peptide (SP), CTD regions of GP64, and TM of HA in the incorporation of HA into the baculovirus envelope. The study demonstrated enhanced HA incorporation and increased HI titers for baculovirus with GP64 SP, CTD, and HA TM. In stark contrast, recombinant baculoviruses containing different combinations of GP64 or HA domains (GP64 SP or HA-SP with HA-TM and HA-CTD; GP64-TM or HA-TM with HA-SP and GP64 CTD; GP64 SP or HA-SP with GP64-TM and GP64-CTD) showed the opposite effect on HA incorporation and HI titers. Based on the results, the second generation of tetravalent baculoviral vaccine was developed using GP64-SP, HA-TM, and GP64-CTD. The vaccine displays HAs from four subclades of H5N1 influenza viruses and induces strong humoral and cellular immunity against homologous and heterologous H5N1 strains [25].

In another study, efficiency of WSSV *ie1* promoter-mediated H5HA expression by recombinant baculovirus was examined [90]. Also, they confirmed that HA0 expressed by the baculovirus was able to successfully translocate to the plasma membrane of the infected cells and observed that surface-displayed HA0 was able to sustain its antigenic conformation and authentic cleavage [91]. It has been reported that baculovirus pseudotyped with vesicular stomatitis virus glycoprotein (VSVG) from vesicular stomatitis virus (VSV) effectively increased transduction levels in mammalian cells [92] and has the capability to increase the amount of expressed protein displayed on the surface of the entire viral envelope [26,93]. In addition, recombinant baculovirus which displays HA and co-expresses VSVG under WSSV *ie1* showed HA displayed on the baculovirus surface has high HA activity. Intranasal or intramuscular immunization of chickens with baculovirus containing WSSV *ie1* induced strong HA-specific antibodies and HI titer compared to baculovirus with *CMV* promoter. Nevertheless, co-expression of VSVG by both viruses contributed to increased anti-HA antibody level and HI activity [27].

Similarly, recombinant baculovirus pseudotyped with VSVG (BV-G-HA) was shown to successfully transduce into mammalian cell, mediate gene delivery, and enable efficient expression of H5HA. An immunization study revealed that the vaccine candidate induced higher levels of H5HA-specific antibodies and cellular immunity compared to the mice vaccinated with 100 µg of DNA vaccine. Furthermore, immunized chickens, administered with varying doses, demonstrated complete protection from a lethal dose of highly pathogenic H5N1 avian influenza virus [26]. However, the vaccine did not efficiently protect mice against an evolutionarily distant strain [26], possibly due to a high degree of genetic divergence of the influenza virus [94]. For example, current monovalent inactivated whole virus vaccine can induce neutralizing antibodies against the homologous strain but shows lower magnitude of response against heterologous strains [95]. Hence, it is necessary to develop a vaccine that expresses antigens covering the major circulating viruses [96]. Therefore, to broaden the protective efficacy of monovalent BV-G-HA vaccine, a pseudotyped baculovirus-based bivalent vaccine that encodes HA from Clade 2.3.4 and Clade 9 influenza viruses was developed. Mice immunization with pseudotyped baculovirus-based bivalent vaccine induced significantly higher humoral and cellular immune responses and provided complete protection against H5N1 viruses from Clade 2.3.4 and Clade 9 compared to other candidates used in the study [97]. Similarly, the cross-protective efficacy of WSSV *ie1* promoter-controlled bivalent baculovirus surface-displayed HA of A/Indonesia/669/06 and A/Anhui/01/05 (bivalent-BacHA) was tested in another study. Mice orally vaccinated using live bivalent-BacHA vaccine showed both systemic and mucosal immunity, unlike mice immunized subcutaneously with live or inactivated bivalent vaccine, which induced only robust systemic immunity. The level of systemic immunity induced by oral bivalent vaccine in immunized mice was lower compared to subcutaneous immunization. However, mice orally immunized with bivalent-Bac-HA vaccine demonstrated strong HA-specific mucosal IgA response. In addition, oral vaccination induced potent cross-clade neutralizing antibodies against distinct clades (1, 2.1.3, 2.2.1.1, 2.3.2, 2.3.4, 4, 7, and 9) of H5N1 viruses which are absent in monovalent inactive whole H5N1 vaccination, whereas mice subcutaneously vaccinated with live or inactivated bivalent-BacHA vaccine showed low neutralization titer against Clade 1 H5N1 compared to other clade of viruses. Enzyme-linked ImmunoSpot assay revealed that mice immunized orally or subcutaneously with live bivalent vaccine triggered both IFN-γ-secreting Th1 cells and IL-4-secreting Th2 cells, while mice immunized subcutaneously with inactive adjuvanted bivalent vaccine stimulated only the IL-4 response. Moreover, mice vaccinated orally or subcutaneously with live bivalent Bac-HA vaccine showed complete cross-protection against Clade 1 and Clade 2.2.1.1 H5N1 viruses, suggesting that this bivalent vaccine can, potentially, protect against cross-clade H5 viruses during pandemic situations [28].

### 2.2. Recombinant Baculovirus Displayed HA Protein of Other Influenza Subtypes and Its Immunogenicity

Since baculovirus has been proved to efficiently express foreign genes and is capable of transduction in a variety of cell lines, researchers utilized this virus as a potential vaccine vector against various infectious agents. Furthermore, additional elements, like VSVG protein, were included by researchers to increase the efficiency of transduction, gene delivery, and expression in the recipient host [92]. While recombinant baculoviral vector expressing both VSV-G and influenza HA was shown to evoke both humoral and cellular immune responses and provided effective protection against lethal virus challenge in mouse and chicken hosts [26], the high cytotoxicity of VSV-G protein [98] and its immediate inactivation by serum complement systems impedes the use of the element in a vaccine delivery vehicle [99]. Recent reports have shown that human endogenous retrovirus (HERV) envelope-coated recombinant baculovirus-based human papillomavirus L1 vaccine significantly improved the expression of L1 in mammalian cells and induced humoral immune responses which are comparable with the level obtained using commercial cervical cancer virus-like particles vaccine [100]. Further, it has been reported that HERV envelope-coated baculovirus-based HA of the 2009 H1N1 pandemic influenza vaccine induced a strong cellular immune response compared to the commercial vaccine [29]. In another study, Prabakaran et al. [101] developed a recombinant baculovirus displayed HA vaccine under the control of WSSV *ie1* promoter (BacHA) against the pandemic 2009 H1N1 virus. The vaccine showed successful HA expression on its envelope, and mice vaccination studies showed that both the live and adjuvanted with inactive form of recombinant baculovirus induced HA-specific antibody responses and offered complete protection against lethal viral infection [101]. Studies have shown that high neutralizing antibodies generated against influenza virus target specific regions in the globular head of influenza HA. Moreover, the HA stalk domain is well conserved and antibodies targeting this region are capable of neutralizing the heterologous influenza viral subtypes [102,103,104,105,106,107]. For example, chimeric HA constructs expressing the globular head and stalk regions from different subtypes induced antibodies that showed broad protection [104,108,109]. Similarly, mice immunized with recombinant baculovirus displayed HA of the 2009 pandemic H1N1 influenza virus induced HA-stalk-specific antibodies and demonstrated protective immunity against homologous and heterologous H5N1 subtype viruses [30].

In recent decades, avian H7 influenza virus was responsible for numerous influenza outbreaks among poultry industries in Europe and North America. Since 1918, none of these poultry-adapted viruses has evolved to widely infect humans or cause a pandemic. However, there are cases where the virus acquired the ability to infect mammals, as evidenced by the largest outbreak of highly pathogenic H7N7 (NL/219.2003) in the poultry industry of the Netherlands, where 89 human infections were reported [110]. H7N7 viruses isolated from the patients were shown to possess replicative potential in human conjunctiva and ability for human-to-human transmission. Interestingly, the virulence of the virus in chickens and turkeys increased with acquisition of additional amino acids at the hemagglutinin (HA) cleavage site [111]. In 2013, human infections with avian influenza A H7N9 subtype were first reported in China and caused several waves of human infection. It was speculated that the virus possesses high mutagenicity, which contributed to its ability to infect humans. Higher mutagenicity may be the cause of enhanced pathogenicity, as supported by recent human cases, where the isolated virus was found to have mutations associated with reduced susceptibility to neuraminidase inhibitors used in antiviral treatment [112]. Since H7 subtype viruses pose a major challenge for public health and the poultry industry, numerous vaccines have been generated against these subtype viruses using various platforms. Recently, baculovirus displayed HA vaccine against H7N7 (NL03) was developed and its efficacy was tested via intranasal (i.n.) or subcutaneous (s.c.) administration in mice. It was observed that intranasally immunized mice with live baculovirus displayed HA vaccine induced higher or comparable humoral, mucosal, and cellular immune responses compared to other vaccine candidates, irrespective of immunization route. Additionally, the immunization provided 100% complete protection, whereas no protection was observed in mice vaccinated with other vaccine candidates [31]. In another study, recombinant baculovirus-based vaccine (BacHA) against avian H7N9 virus with high pandemic potential was generated, and its immunogenicity and cross-protective efficacy was evaluated in mice. They found that live BacHA i.n. immunized mice elicited high levels of HA-specific IgA mucosal immune response and humoral immune response, comparable to mice administered subcutaneously with live BacHA. On the other hand, subcutaneous immunization with adjuvanted inactive BacHA stimulated robust humoral immune response and induced higher neutralizing antibodies against H7N9 viruses and other H7 subtype (H7N7 and H7N3) viruses compared to other vaccine candidates. In addition, subcutaneous immunization with inactive whole H7N9 vaccine protected 100% against RG-H7N9 challenge but demonstrated insufficient protection against RG-H7N7 (NL03) subtype, whereas mice vaccinated subcutaneously with live or inactive recombinant baculovirus against H7N7 (NL03) protected only 33–50% of mice against H7N9 challenge. Furthermore, mice immunized i.n. with live BacHA of H7N7 or H7N9 showed complete protection against both H7N7 and H7N9 infection in a challenge study [32]. It has been reported that the differences between the immunogenicity of HA of H7N7 (NL03) and HA of H7N9 might be due to the amino acid changes at A143T, which introduced a potential glycosylation site at position 141N, masking the epitopes of H7HA (NL03) [113,114]. To account for this difference in immunogenicity, Kumar et al. [115] compared the HA of H7N9 and H7N7 viruses and selected three positions in their study: (a) 143, (b) 198, and (c) 211, which are located within or near the receptor-binding site of H7N7 HA protein. By site-directed mutagenesis, they generated six mutant constructs by single or double amino acid substitution at these three positions (T143A, T198A, I211V, T143A-198A, T198A-I211V, and I211V-T143A). Among the generated mutant constructs (Bac-HA_m_), mice vaccinated with constructs T143A, T198A-I211V, and I211V-T143A induced higher hemagglutination inhibition and neutralization titer that neutralizes both H7N7 and H7N9 viruses compared to mice immunized with BacHA or other mutant vaccines. The result shows that the substitution of amino acids responsible for forming potential glycan motif-epitope masks would improve immunogenicity of the vaccine candidates. In another study, mice i.n. immunized with baculovirus-displaying HA of low pathogenic influenza H6 subtype triggered higher HA-specific humoral, mucosal, and cellular immunity compared to mice immunized with inactivated influenza virus. Moreover, regardless of immunization route, it conferred 100% protection against lethal homologous mouse-adapted H6N1 virus [33]. Moreover, recombinant baculovirus with CMV-polyhedrin dual promoter for expressing chimeric HA of H9N2 was shown to efficiently express HA in both mammalian and insect cells, induce strong immune response, and provide 100% protection against lethal H9N2 viral challenge in mice, unlike other vaccine candidates observed [34].

## 3. Recombinant Baculovirus Display of Other Viral Proteins and Its Immunogenicity

Shrimp immunity relies on an innate immune mechanism, and their lack of adaptive immunity hinders vaccine development against shrimp pathogens. Though limitations exist in developing shrimp vaccines, the presence of quasi-immune responses after natural or experimental infection with white spot syndrome virus (WSSV) in *P. japonicas* and the resistance against experimental rechallenge with WSSV due to neutralizing factors in the survivors’ plasma [116,117] encouraged the researchers to continue the development of vaccines against the WSSV virus. Several attempts have been made to develop vaccines by targeting the immunogenic VP28 protein of WSSV virus [118,119,120,121] Syed Musthaq et al. [122], developed a recombinant-based vaccine (Bac-VP28) that displays recombinant VP28 (rVP28) on its envelope. Confocal and transmission electron microscopy analysis confirmed that rVP28 was able to localize on the plasma membrane of infected insect cells, and the virus also successfully acquired rVP28 on its envelope from the insect cell membrane during the budding process. Approximately 65.3 µg/mL of VP28 protein was anchored on the Bac-VP28 envelope (10^8^ viral particles). Furthermore, its protective efficacy against WSSV infection was tested via injection, oral, or immersion methods in shrimp. The Bac-VP28-injected shrimp successfully transcribed and translated VP28 and showed survival rates of 86.3% and 73.5% against WSSV virus challenge at 3 and 15 days postvaccination, respectively [122]. On the other hand, the transcriptional levels of lipopolysaccharide and β-1,3-glucan binding protein (LGBP) and signal transducer and activator of transcription (*STAT*) genes (which are crucial for triggering innate immune responses) [123] were altered at different days postinfection (dpi) in shrimp vaccinated with Bac-VP28 by oral or immersion routes. The expression level of *STAT* gene in the control group was downregulated at various dpi as WSSV infection progressed, but in the vaccinated group, *STAT* gene level decreased until 7 dpi and started to increase at 10 dpi and 15 dpi. Moreover, the levels of *LGBP* gene in the control group was upregulated as infection progressed, whereas the vaccinated group showed an increasing level at early infections, which later decreased as WSSV infection cleared. These results confirm that oral vaccination of Bac-VP28 triggers the innate immune responses and, possibly, was responsible for higher survival rates of 81.7% and 76.7% upon WSSV challenge at 3 and 15 days postvaccination. Also, shrimp vaccinated with Bac-VP28 immersion vaccine showed 75% and 68.4% survival rates compared to 100% mortality obtained with the control group in a challenge study [35].

Porcine reproductive and respiratory syndrome virus (PRRSV), classical swine fever virus (CSFV), and porcine circovirus type 2 (PCV2) are the major pathogens continuously affecting the pig industry and cause significant economic losses worldwide. To date, researchers all over the world have contributed various forms of vaccines utilizing different strategies targeting these pathogens. Virus-based vectored vaccines were developed using modified vaccinia virus Ankara, canine adenovirus-2, pseudorabies virus, and Semliki Forest virus [124,125,126,127]. However, these vectors are of mammalian origin and pose a biohazard threat. In contrast, recombinant baculoviruses possess good safety profiles and have been utilized to develop vaccines against PRRSV, CSFV, and PCV2 viruses. Using GP64 SP, TM, and CT domains, Xu et al. [36] developed recombinant baculovirus-based bivalent vaccine vector (BacSC-Dual-GP5-Cap) that surface-displayed GP5 (major immunogenic inducer of neutralizing antibodies against PRRSV infection) and PCV2 (major immunogenic capsid (cap) protein). A swine vaccination study demonstrated that this vaccine candidate stimulated potent GP5 and cap-specific neutralizing antibodies and induced higher lymphocyte proliferation responses compared to control groups, which indicates that the bivalent vaccine can potentially be used as a vaccine against a mixed PRRSV and PCV2 infections. In another study, the ORFs of PRRSV were successfully displayed on the baculovirus envelope using the GP64 SP, H3N2 TM, and GP64 CTD regions. The antibody responses elicited by the vectored vaccines were sustained for up to 30 days after the booster, whereas the titers in the inactivated PRRSV-vaccinated group were reduced dramatically at this time point. The enhanced immune responses could be due to the in vivo transduction of the PRRSV genes by baculovirus vaccine, which activates the endogenous antigen-processing and -presenting pathways. Amongst the vaccines developed in this study, baculoviruses that displayed ORF2a, ORF3, and ORF4 induced higher antibody responses in mice compared to other vaccine candidates [37]. A recombinant baculovirus-based vaccine that contains the PCV2 cap and VSVG protein (BV-GD-ORF2) was developed. The expression of PCV2 was placed under the control of *CMV* and *polh* promoters, whereas expression of VSVG was placed under the *p10* promoter. Due to VSVG-mediated transduction and the successful expression of the PCV2 cap protein in transduced cells, BV-GD-ORF2 behaved as a subunit/DNA vaccine and induced robust cap protein-specific humoral and cellular immune responses [38]. Studies reported that E2 or E^rns^ glycoprotein are the main immunogenic proteins of CSFV, which can induce neutralizing antibodies and provide protective immunity against CSFV. Xu et al. generated recombinant baculoviruses that surface-displayed either E2 (BacSc-E2) [39] or E^rns^ (BacSc-E^rns^) [40] as fusion forms with GP64 domains. The presence of the GP64 domains did not alter the viral titers of the recombinant viruses. Moreover, a mice vaccination study revealed that BacSc-E2 or BacSc-E^rns^ induced stronger E2- or E^rns^-specific antibodies, respectively, compared to other vaccine candidates, suggesting that the vaccine candidates could potentially be applied in the treatment of CSFV infection.

Respiratory syncytial virus (RSV) is a common contagious virus that causes acute lower respiratory tract infection in infants, young children, elderlies, and immunocompromised individuals [128]. Currently, there is no approved vaccine available to treat this infection in humans. Studies have reported that immunization with recombinant viral vector expressing RSVG protein showed immune pathological issues by enhancing Th2 skewing of the CD4^+^ T-cell response [129,130,131]. Countering the issue, Zhang et al. [41] developed recombinant baculovirus vaccines that showed reduced immune pathological conditions in mice. Four vaccine constructs were developed: Bac-tF64 (displays the F ectodomain (tF) on the envelope), Bac-CF (expresses full-length F protein in transduced mammalian cells), Bac-CF/tF64 (displays tF on the envelope and expresses F in cells), and Bac-CF/tF64-VISA (contains additional virus-induced signaling adaptor (*VISA* gene). Bac-CF and Bac-CF/tF64 showed an increased mixed Th1/Th2 cytokine response, higher levels of IgG2a/IgG1 antibody responses, and reduced immunopathology upon RSV challenge compared to Bac-tF64. Moreover, co-expression of VISA reduced the Th2 immune responses induced by Bac-CF/tF64 and protected lungs from inflammatory immune response upon a subsequent RSV challenge. Therefore, the inclusion of *VISA* elements could be an effective alternate vaccine strategy against RSV infection [41].

Rabies virus (RABV) is highly infectious and poses a constant threat to humans and animals. Traditionally, vaccine development against RABV mainly focused on inducing neutralizing antibodies [132,133,134]. However, studies have shown that, in addition to humoral immunity, cellular immune responses are important for providing protective immunity against RABV [42,135,136]. Previously, a recombinant baculovirus viral vector vaccine against RABV glycoprotein (RVG) was constructed (BV-RVG/RVG). The vector expresses two types of RVG antigen (baculovirus-expressed RVG and in vivo expressed RVG) to enhance specific immune responses against the RVG antigen. Based on immunization study, BV-RVG/RVG-immunized mice displayed higher humoral and cellular immune responses and offered complete protection against RABV compared to mice immunized with other vaccine candidates. The enhanced immunogenicity could be due to immediate recognition of RVG incorporated into BV-RVG/RVG by immunocompetent cells, resulting in presentation to dendritic cells (DCs) and, subsequently, activation of B cells to secrete viral-specific neutralizing antibodies. Furthermore, *CMV*-mediated in vivo RVG expression by BV-RVG/RVG activated the endogenous antigen presentation pathway, inducing persistent and specific immune responses [42]. In another study, Feng et al. [43] displayed the entire ectodomain of SARS-CoV spike protein (S) on the baculovirus envelope using GP64 signal peptide and VSV-G membrane anchor. In mice, the vaccine induced specific neutralizing antibodies against S protein in the absence of adjuvants [43].

In a subsequent study, researchers developed recombinant baculovirus vaccines against avian infectious bronchitis virus (IBV), Japanese encephalitis virus (JEV), and bovine herpesvirus-1 (BHV-1) with the GP64 domains (SP, TM, and CTD). Chickens immunized with baculovirus displayed S1 protein of IBV vaccine induced more humoral and cellular immune responses and showed better protection (83%) compared to other vaccines studied [44]. On the other hand, glycoprotein E of JEV displayed on the baculovirus envelope induced strong neutralizing antibodies in mice and swine and offered 100% protection in vaccinated mice against a lethal JEV challenge [45]. Similarly, BHV-1 envelope glycoprotein (gD) displayed on the baculovirus envelope elicited strong neutralizing antibody against BHV-1 in mice [46].

## 4. Recombinant Baculovirus Display of Viral Capsid Proteins and Its Immunogenicity

Enterovirus 71 (EV71) immunogenic capsid protein (VP1) is a non-membrane protein which requires additional regions, like SP, TM, and CTD of GP64, HA, or other domains for successful incorporation into envelope, while membrane proteins like HA or NA (neuraminidase) can be translocated to the plasma membrane and incorporated onto the envelope during viral budding. Meng et al. [47] generated recombinant baculoviruses, with WSSV *ie1* (Bac-Pie1-gp64-VP1) or polyhedrin promoter (Bac-Pph-gp64-VP1), which surface-displayed chimeric VP1 (VP1 was fused in between GP64 SP and mature domain). Transmission electron microscopy analysis confirmed that VP1 was successfully anchored on the recombinant baculoviral envelope. Bac-Pie1-gp64-VP1-immunized mice were found to induce slightly higher VP1-specific antibodies with potent neutralizing activity (can neutralize C4 subgenogroups and heterologous subgenogroups) than Bac-Pph-gp64-VP1 and to show neutralizing activity comparable with inactivated EV71 vaccine. Subsequently, Premanand et al. [137] generated the recombinant baculovirus (Bac-VP1) that surface-displayed the chimeric VP1 using minimal fusion partners, like GP64 SP (20 aa), H3N2-HA transmembrane domain (27 aa), and GP64 CTD (6 aa) to facilitate better incorporation of VP1 and higher baculovirus titers. Expectedly, Bac-VP1 contains 3-fold higher VP1 (39 ng/µg) on its envelope compared to VP1 on Bac-Pie1-gp64-VP1 envelope (13 ng/µg). Bac-VP1-immunized mice elicited stronger VP1-specific antibody responses and cross-neutralization activity against homologous or heterologous strains compared to Bac-Pie1-gp64-VP1 and Bac-Pph-gp64-VP1 vaccines. Moreover, Bac-VP1 provided 100% protection against a mouse-adapted lethal strain of EV71 [137]. In both studies mentioned above, VP1 was anchored on the viral envelope similar to type 1 transmembrane protein with unrestricted N-terminus exposure. Mimicking other types of transmembrane protein, Kolpe et al. generated recombinant baculovirus (Bac-NA-VP1) which surface-displayed VP1 using influenza A neuraminidase (NA), resulting in a type II transmembrane-anchored pattern with a distal C-terminus. Mice immunization studies revealed that Bac-NA-VP1 induced both humoral and cellular immune responses and provided 100% protection of suckling Balb/c mice against mouse-adapted EV71-B4 strain challenge [48].

Infectious bursal disease virus (IBDV) is the causative agent of infectious bursal disease, which affects chickens and causes considerable economic loss for the poultry industry [138,139]. It has been reported that live attenuated vaccines elicited lower level of antibodies and failed to protect the chickens against IBDV [140]. To target this issue, Xu et al. [49] generated a recombinant baculovirus that surface-displayed His6-tagged VP2 protein using the GP64 TM and CT domains (BacSc-VP2). BacSc-VP2-immunized chickens demonstrated higher levels of neutralizing antibodies and better protective immunity against a very virulent IBDV strain than control groups. Furthermore, IBDV-specific lymphocytes were observed in BacSc-VP2-vaccinated chickens. Also, the major immunogenic capsid protein VP2 of canine parvovirus type 2 (CPV-2) and immunogenic proteins σC and σB of avian reovirus (ARV) have been utilized to develop a baculovirus-based vaccine against CPV-2 and ARV, respectively. Fusion of GP64 domains (SP, TM, and CTD) with the capsid proteins allows their incorporation into the baculoviral envelope. An animal vaccination study revealed that baculovirus that surface displayed VP2 of CPV-2 induced higher neutralizing antibodies in mice compared to other vaccines tested [50]. Moreover, recombinant baculoviruses that displayed immunogenic proteins of either σC or σB of ARV elicited strong antibody titers with higher neutralizing activities compared to antibody responses by other vaccine candidates [51].

## 5. Recombinant Baculovirus Display of Malarial Parasite Proteins and Its Immunogenicity

Malaria, a global disease caused by *Plasmodium* parasites, is endemic in tropical and subtropical regions and results in up to 1 million deaths annually. A recombinant protein-based subunit vaccine containing a part of a major surface protein from *Plasmodium falciparum* circumsporozoite protein (PfCSP) provided short-lived protective immunity, and the effective period was affected by age group and malaria transmission intensity [141,142]. Another vaccine *Plasmodium falciparum* sporozoite (PfSPZ) was reported to provide protection against homologous and heterologous human malaria infection [143,144,145]. Aiming to curb the spread of malaria, the WHO has set to a goal of 80% vaccine efficiency by the year 2025. Novel and alternate strategies will be required to ensure the development of more effective vaccines against malaria infection. To develop a vaccine with dual functions (such as a malaria vaccine that can act as a liver-directed gene delivery vehicle), Yoshida et al. [59] developed the recombinant baculovirus-based vaccine that displayed rodent malaria parasite *Plasmodium berghei* circumsporozoite protein (PbCSP). In the immunization study, the vaccine induced both humoral and cellular immune responsea and provided 60% protection against sporozoite challenge [59]. Subsequently, a series of baculovirus-based vaccines expressing PfCSP (with deleted glycosylphosphatidylinositol GPI anchor region) under the control of *polh* or *CMV* promoter was developed. AcNPV.CS vector expresses the CS protein in APCs under the control of the *CMV* promoter to enable (preferentially) induction of CD8^+^ T-cells upon MHC Class I presentation. AcNPV.CS64 vector expresses a CS protein with GP64 as a fusion form using the polyhedrin promoter in insect cells, which allows the incorporation of CS on the baculovirus envelope during the budding process (mimicking the *P. falciparum* sporozoite). Uptake of AcNPV.CS64 into APCs triggers CD4^+^ cells via MHC Class II presentation and induces a potent humoral immune response. AcNPV.CS-CS64 combines expression and presentation of recombinant CS antigens in mammalian cells to induce both CS-specific CD4^+^ and CD8^+^ T-cells. A mouse vaccination study showed that the AcNPV.CS-CS64 vaccine induced efficient humoral and cellular immune responses with intramuscular immunization compared to other vaccine candidates [60]. Recombinant baculovirus dual expression-based malaria vaccine, which drives PbCSP expression by *polh* and *CMV* promoters, was developed. Mice immunized with the vaccine induced both Th1 and Th2 responses after intramuscular inoculation and provided complete protection against sporozoite challenge [58]. Another baculovirus-based vaccine candidate which displays the major surface antigen of *P. falciparum*, pPfs25, was developed as a transmission-blocking vaccine against human malaria. Both i.m. and i.n. immunized mice showed high levels of Pfs25-specific antibodies and effective transmission-blocking response, with an 83% (intranasal) and 95% (intramuscular) reduction in oocyst intensity [55]. In another case study, a recombinant baculovirus-based *Plasmodium vivax* transmission-blocking vaccine using *Autographa california* nuclear polyhedrosis virus (AcNPV-Dual-Pvs25) was developed. AcNPV-Dual-Pvs25 not only displayed Pvs25 on the AcNPV envelope in native conformation, but also expressed Pvs25 upon transduction of mammalian cells. AcNPV-Dual-Pvs25 induced Pvs25-specific antibodies in intranasally or intramuscularly immunized mice. Moreover, in a rabbit immunization study, the vaccine induced significant transmission-blocking response, regardless of immunization route (subcutaneous, intramuscular, and intranasal) [56]. Yoshida et al. [57] developed a baculovirus-based nasal drop vaccine (AcNPV-PyMSP1_19surf_) that surface-displayed the carboxyl terminus of merozoite surface protein 1 (PyMSP1_19_) from *Plasmodium yoelii.* Intranasal and intramuscular immunization of mice with AcNPV-PyMSP1_19surf_ induced mixed Th1/Th2 immune responses and provided 100% protective efficacy. However, intranasal immunization elicited higher PyMSP1_19_-specific antibody titers and stronger natural boosting after a challenge study [57]. In another study, a baculovirus dual-expression system-based PfCSP protein (BDES-PfCSP) vaccine was generated. Based on an immunization study, the vaccine was found to induce mixed Th1/Th2 immune responses in mice, generate a higher level of PfCSP-specific antibodies, and confer significant protection against malaria. In addition, sera from immunized monkeys prevented sporozoite invasion in HepG2 cells [53]. A multistage malaria vaccine was developed using the baculovirus platform to target and inhibit the *Plasmodium* parasite in its pre-erythrocytic and sexual stages. The generated baculovirus displayed correct folding of both *P. vivax* circumsporozoite protein (PvCSP) and P25 (Pvs25) protein in fusion form (BDES-Pvs25-PvCSP) and acted as a pre-erythrocytic and a transmission-blocking activity vaccine. The BDES-Pvs25-PvCSP vaccine induced higher antibody levels against Pvs25 and PvCSP while providing 43% protection against malaria and 82% transmission-blocking activity [54]. In a recent study, human decay-accelerating factor (hDAF) was incorporated into a baculovirus vector, which effectively displayed hDAF and PFCSP on the surface of the baculoviral envelope to prevent serum complement inactivation. Following intramuscular immunization, the modified vaccine conferred higher protective efficacy (60%) compared to control group (30%) in a challenge study [52].

## 6. Development of Baculovirus Surface-Displayed Mucosal Vaccines for Broad Protection

Mucosal surfaces (respiratory, gastrointestinal, and urogenital tracts) provide the major route of entry for various microorganisms, and the mucosal immune system acts as first line of defense against microbial infection [146]. Therefore, triggering of the mucosal immune system is important in preventing infection, and this can be achieved by mucosal vaccinations via intranasal, oral, pulmonary, rectal, or vaginal routes of administration. Among these routes, oral or intranasal immunizations are the two most viable options for vaccine delivery. The nasal route, in particular, offers several advantages, like the possibility of self-administration, the induction of both mucosal and systemic immunity in the gastric mucosa, respiratory tracts and genital tracts, as well as the lower doses of antigen and adjuvant required compared with oral vaccination. Several nonhuman viral vectors have been developed in recent years, among which is the baculoviral vector. Baculovirus has been reported to possess strong adjuvant properties in mammals, promoting enhanced humoral, mucosal, and cellular immune responses against co-administered antigens. Supporting this are previous reports which indicated that mice immunized with baculovirus genomic DNA and ovalbumin showed humoral and cytotoxic T-lymphocyte responses against co-administered antigens. The abundance of CpG motifs in the baculovirus improves its ability to induce innate immune responses through the TLR9/MyD88-dependent endosomal recognition pathway and DNA-dependent activator of interferon regulatory factor-mediated pathway [147]. Other studies have shown that intranasal inoculation of mice with wild-type baculovirus provided protection against H1N1, H3N2, and other influenza B strains [148], which highlighted the potential of the baculoviral vector in the development of vaccines against mucosal acquired pathogens. Intranasal immunization of mice with live recombinant baculovirus (Bac-HA) vaccine induced significantly higher mucosal IgA immune response against H7N7-HA compared to other intranasally delivered vaccines. The higher immune response could be attributed to the efficient transduction of baculoviral vector or the strong adjuvant property of baculovirus due to the presence of unmethylated CpG motifs. In addition, i.n delivery of Bac-HA stimulated higher amounts of IFN-γ and IL-4 compared to s.c immunization of live Bac-HA, which is similarly reported in other studies [149,150]. The enhancement of cytokine levels could be due to recall response in splenocytes or due to the presence of nasopharynx-associated lymphoid follicles in the nasal mucosa that trigger antigen-specific Th lymphocytes, CTLs, and IgA immune responses. A challenge study in mice revealed that i.n immunized mice with live Bac-HA showed 100% protection against lethal viral challenge compared to other vaccines, irrespective of route of administration. Even though s.c immunized mice achieved a higher level of IgG antibodies, the incapability of Bac-HA in protecting against lethal viral challenge could be due to lack of mucosal IgA, which is more crucial in clearing upper respiratory tract infection than IgG [31]. In another study, a recombinant baculovirus-based vaccine against another pandemic potential avian H7N9 virus was generated (Bac-HA). They found that mice intranasally immunized with live Bac-HA elicited higher levels of HA-specific mucosal and humoral immune responses. Mice vaccinated subcutaneously with live or inactive baculovirus displayed HA of H7N7 (NL03) only protected 33–50% of mice against H7N9 challenge, but mice immunized intranasally with live Bac-HA of H7N7 or H7N9 showed complete protection against both H7N7 and H7N9 infection in a challenge study. The result indicates the importance of additional mucosal immunity in recovery and protection during H7 subtype infection [32]. The inclusion of adjuvant improves the immune response induced by the vaccine, and the cause of the better immune response in mucosal adjuvants, in particular, is due to the induction of protective local and systemic immune responses against various infectious diseases [151]. Also, intranasal immunization of recombinant baculovirus-displaying HA of H5N1 virus induced high levels of mucosal IgA and serum IgG immune responses in mice. Combination of recombinant mucosal adjuvant CTB further enhanced serum IgG and IgA responses compared to unadjuvanted log 2^4^ or log 2^8^ HA titer Bac-HA alone. Log 2^8^ HA titer live BacHA with 10 µg of rCTB conferred 100% protection against homologous and heterologous H5N1 strains [90], suggesting that the combination of safe adjuvants with vaccines would be ideal for optimal efficacy of intranasal vaccines.

Oral administration is considered as another efficient route for delivering the mucosal vaccines. It is non-invasive, safe, and shows good patient compliance. For example, oral polio vaccine against highly contagious poliovirus helped to eradicate poliovirus infection in most parts of the world [152]. Moreover, studies have shown that oral vaccination can prevent infection of lungs and is capable of inducing mucosal immune responses (IgA) in the respiratory tract, providing protection against influenza viruses. Prabakaran et al. [91] reported that live recombinant baculovirus displaying HA of H5N1 virus (Bac-HA) was able to express HA in epithelial cells of intestines of orally vaccinated mice and induced strong systemic and mucosal immune responses. Mice immunized with live Bac-HA of H5N1 without any inclusion of adjuvants conferred 100% protection against homologous and heterologous H5N1 strains, while immunization with inactivated Bac-HA resulted in a lower survival rate of 33.3%. The reduced immune responses of the inactivated Bac-HA could be due to loss of transduction ability, whereas the live Bac-HA was able to bind to the receptors that are expressed in the intestinal cells, resulting in gene delivery and stimulation of cell-mediated immune responses [91]. Moreover, the formulation of inactivated vaccine can have a huge impact on its immunogenicity. In one study, the immunogenicity lost by inactivated baculovirus displayed HA (Bac-HA) vaccine was recovered by encapsulation using reverse micelle of phosphatidylcholine adjuvant. Mice orally immunized with encapsulated inactive Bac-HA induced systemic and mucosal immune response comparable to live Bac-HA [153]. However, there are limitations in the use of mucosal adjuvants in humans. Aside from effectiveness of the adjuvant, the major concern in using mucosal adjuvants is its possible detrimental impact on humans. Therefore, adjuvant used in mucosal immunization has to be proven safe for use. Premanand et al. [61] utilized naturally occurring lipid-based vesicles (bilosomes) as a mucosal adjuvant to coat the recombinant baculovirus (Bac-VP1). An oral immunization study revealed that Bac-VP1, associated with bilosomes, induced higher VP1-specific immune responses and conferred complete protection in a challenge study [61]. To overcome the hurdles of selecting safe mucosal adjuvants, data analysis can be applied to search for suitable mucosal adjuvants and to understand their mechanism of action. In addition to new technologies, novel concepts are welcomed in the development of suitable mucosal adjuvants. Finally, considerations on the delivery route and the appropriate combination of vaccine and adjuvant are essential in ensuring optimal effectiveness of vaccination in humans.

## 7. Conclusions and Future Perspective

Baculovirus surface-displayed vaccines have been proven effective in inducing protective immune responses in animal models. However, further advancement in the context of immunogenicity will be made by utilizing hybrid promoters for the enhanced expression and anchoring of complex protein on recombinant baculoviral envelopes. In addition, inclusion of novel regulatory elements and molecular adjuvant property-carrying motifs in the vector will certainly boost the immunogenicity of antigen displayed on the baculovirus envelope. The main obstacle in implementing baculovirus surface-displayed vaccine as a human vaccine candidate is the lack of safety guidelines for human immunization. However, international organizations, like Organization of Economic Co-operation and development in 2002 [154] and the European Commission’s Health & Consumer Protection Directorate-General in 2008 [155], concluded that no adverse effects of baculovirus on human health have been observed in safety tests, and they are not pathogenic, carcinogenic, or genotoxic in mammalian cells. However, environmental and ecological impacts of the baculovirus in invertebrates will be reduced by modification of baculovirus essential genes required for replication and budding, allowing the development of a baculovirus surface-displayed vaccine with an optimal safety profile in the future.

## Figures and Tables

**Table 1:** Recombinant baculovirus displayed antigens against infectious diseases.

Recombinant Baculovirus	Target Antigen	Immunized Species	Promoters	Induced Immune Responses			References
				Humoral	Cellular	Mucosal	
Bac-CHA/HA64	HA (H5)	Mice	*Pp10*, *P_CMV_-IE*	Yes	Yes	Yes	[20]
BV-Dual-3M2e-LTB	HA (H5)	Mice	*Pp10*, *P_CMV_-IE, P_PH_*	NT	Yes	Yes	[21]
Bac-HAMW	HA (H5)	Mice	*Pp10*, *P_CAG_*	Yes	Yes	NT	[22]
BV-A-ITRs-HA	HA (H5)	Chickens	WSSV-*ie1*	Yes	Yes	NT	[23]
vAc-HA-DUAL	HA (H5)	Mice	*P_PH_*, *CMV*, *Pp10*	Yes	Yes	Yes	[24]
Bac-spHAct	HA (H5)	Mice	*P_PH_*	Yes	Yes	NT	[25]
BV-G-HA	HA (H5)	Mice Chickens	*P_PH_*, *CMV*	Yes	Yes	NT	[26]
vAC-ie-HA	HA (H5)	Chickens	WSSV-*ie1*	Yes	NT	NT	[27]
BacHA	HA (H5)	Mice	WSSV-*ie1*	Yes	Yes	Yes	[28]
AcHERV-sHINI-HA	HA (H1)	Mice	*P_PH_*	Yes	Yes	NT	[29]
rBac-HA	HA (H1)	Mice	*P_PH_*	Yes	Yes	Yes	[30]
Bac-HA	HA (H7)	Mice	WSSV-*ie1*	Yes	Yes	Yes	[31]
BacHA	HA (H7)	Mice	WSSV-*ie1*	Yes	NT	Yes	[32]
Bac-HABV-Dual-HA	HA (H6) HA (H9)	Mice Mice	WSSV-*ie1* *P_PH_*, *CMV*	Yes Yes	Yes NT	Yes NT	[33] [34]
Bac-VP28	WSSV-VP28	Shrimp	WSSV-*ie1*	NT	NT	NT	[35]
BacSC-Dual-GP5–Cap	GP5 (PRRSV), Cap (PCV2)	Swine	*Pp10*, *P_PH_*	Yes	Yes	NT	[36]
Bac-ORF2a, ORF4	ORF2a, ORF4 (PRRSV)	Mice	WSSV-*ie1*	Yes	NT	NT	[37]
BV-GD-ORF2	ORF2 (PCV2)	Mice	*Pp10*, *P_PH_, CMV*	Yes	Yes	NT	[38]
Bac-SC-E2	E2 (CSFV)	Mice	*Pp10*, *P_PH_, CMV*	Yes	NT	NT	[39]
BacSc-E^rns^	E^rns^ (CSFV)	Mice	*Pp10*, *P_PH_, CMV*	Yes	NT	NT	[40]
Bac-CF/tF64-VISA	F protein (RSV)	Mice	*Pp10*, *CMV*	Yes	Yes	NT	[41]
BV-RVG/RVG	RVG (RABV)	Mice	*P_PH_*, *CMV*	Yes	Yes	NT	[42]
S-vsvG	Spike (s) protein (SARS-CoV)	Mice	*P_PH_*	Yes	NT	NT	[43]
BV-Dual-S1	S1 protein (IBV)	Chickens	*P_PH_*, *CMV*, *Pp10*	Yes	Yes	NT	[44]
BacSC-E	E protein (JEV)	Mice/Swine	*P_PH_*, *CMV*, *Pp10*	Yes	Yes	NT	[45]
AcSupgD	D protein (BHV-1)	Mice	*P_PH_*	Yes	NT	NT	[46]
Bac-Pie1-gp64-VP1	VP1 (EV71)	Mice	WSSV-*ie1*	Yes	NT	NT	[47]
Bac-NA-VP1 BacSc-VP2	VP1 (EV71) VP2 protein (IBDV)	Mice Chickens	WSSV-*ie1* *P_PH_*, *CMV*, *Pp10*	Yes Yes	Yes Yes	NTNT	[48] [49]
BacSC-VP2	VP2 (CPV-2)	Mice	*P_PH_*, *CMV*, *Pp10*	Yes	NT	NT	[50]
BacSC-σC, BacSC-σB	σC, σB protein (ARV)	Mice	*P_PH_*, *CMV*, *Pp10*	Yes	NT	NT	[51]
BDES-sPfCSP2-Spider	PfCSP (*P. falciparum*)	Mice	*P_CMV_-IE*, *P_PH_*	Yes	NT	NT	[52]
BDES-PfCSP	PfCSP (*P. falciparum*)	Mice/Monkeys	*P_CMV_-IE*, *P_PH_*, *CAG*	Yes	Yes	NT	[53]
BDES-Pvs25-PvCSP	PvCSP (*P. vivax*)	Mice	*P_CMV_-IE*, *P_PH_*	Yes	Yes	NT	[54]
AcNPV-Pfs25surf	Pfs25 (*P. falciparum*)	Mice	*P_PH_*	Yes	NT	NT	[55]
AcNPV-Dual-Pvs25	Pvs25 (*P. vivax*)	Mice/Rabbit	*P_CMV_-IE*, *P_PH_*	Yes	NT	NT	[56]
AcNPV-PyMSP1_19surf_	PyMSP1_19_ (*P. yoelii*)	Mice	*P_PH_*	Yes	NT	NT	[57]
AcNPV-Dual-PbCSP	PbCSP (*P. berghei*)	Mice	*P_CMV_-IE*, *P_PH_*	Yes	Yes	NT	[58]
AcNPV-CSPsurf	PbCSP (*P. berghei*)	Mice	*Pp10*, *P_PH_*	Yes	Yes	NT	[59]
AcNPV.CS-CS64	CS (*P. falciparum*)	Mice	*P_CMV_-IE*, *P_PH_*	Yes	Yes	NT	[60]
Bac-VP1	VP1 (EV71)	Mice	WSSV-*ie1*	Yes	NT	Yes	[61]

NT—Not tested.
